# Immunoelectron Microscopy of Viral Antigens

**DOI:** 10.1002/cpmc.86

**Published:** 2019-06-20

**Authors:** Neetu M. Gulati, Udana Torian, John R. Gallagher, Audray K. Harris

**Affiliations:** ^1^ National Institute of Allergy and Infectious Diseases, National Institutes of Health Bethesda Maryland

**Keywords:** antigen detection, immunogold labeling, influenza virus, transmission electron microscopy, vaccines, viral glycoproteins, viruses

## Abstract

Immunoelectron microscopy is a powerful technique for identifying viral antigens and determining their structural localization and organization within vaccines and viruses. While traditional negative staining transmission electron microscopy provides structural information, identity of components within a sample may be confounding. Immunoelectron microscopy allows for identification and visualization of antigens and their relative positions within a particulate sample. This allows for simple qualitative analysis of samples including whole virus, viral components, and viral‐like particles. This article describes methods for immunogold labeling of viral antigens in a liquid suspension, with examples of immunogold‐labeled influenza virus glycoproteins, and also discusses the important considerations for sample preparation and determination of morphologies. Together, these methods allow for understanding the antigenic makeup of viral particulate samples, which have important implications for molecular virology and vaccine development. © 2019 The Authors. This is an open access article under the terms of the Creative Commons Attribution License, which permits use, distribution and reproduction in any medium, provided the original work is properly cited.

## INTRODUCTION

Immunoelectron microscopy is founded upon the principles of transmission electron microscopy (TEM). TEM is an imaging technique that relies on a high‐voltage electron beam being passed through a specimen, resulting in an image created from the interaction between the electrons and the sample. Electron microscopes are capable of much higher resolution than light microscopes because the effective wavelength of the electron beam is 10^5^‐fold shorter than the wavelength of visible light. Indeed, the resolution of a TEM microscope is limited not by the wavelength of an electron, but by the optics required to focus the beam and by the stability of the sample. The electron beam requires high vacuum to traverse the distance inside the TEM column, demanding that a liquid sample be dried to the grid using an electron‐scattering stain to provide contrast for imaging or flash‐frozen in vitreous ice after application to the grid for cryomethods (Gulati, Pitek, Steinmetz, & Stewart, 2017).

Negative staining is a quick and accessible way to identify structural information about a sample of particles in suspension that avoids many of the challenges associated with cryomethods, including cost and time investment. Immunoelectron microscopy uses negative staining techniques while allowing for antigen detection and for determining structural organization of samples. This is especially helpful for ambiguous samples in which the presence of a particular antigen is unknown or if multiple conformations prevent identification and localization. Often samples prepared for immunoelectron microscopy are ultrathin sections of tissue treated for microscopy applications, but valuable information can also be obtained from immunogold labeling of liquid samples of viral suspensions. Not only can these techniques be applied to studying live viruses, but they are also useful for characterizing viral components in vaccines, viral‐like particles, proteins purified from viruses, and other specimen relevant in modern virology applications (Gallagher et al., 2018; Lynch, Meyers, Williamson, & Rybicki, 2012). Immunoelectron microscopy has also been applied to study many other molecular biology and nanotechnology specimens (Bruckman, Randolph, Gulati, Stewart, & Steinmetz, 2015; Geuze et al., 1984; Zuber, Spiro, Guhl, Spiro, & Roth, 2000).

This article outlines procedures for immunogold labeling of vaccines and viral suspensions to identify and investigate the structural localization and organization of antigens of interest. Other considerations are also discussed, including how to prepare a viral sample for immunogold labeling (Support Protocol 1) and identifying basic structural morphology through negative staining (Support Protocol 2).

## IMMUNOELECTRON MICROSCOPY STAINING USING GOLD‐CONJUGATED SECONDARY ANTIBODIES

The following protocol describes the basic steps in immunolocalization of antigenic components of a viral particulate sample. This technique allows for detection of particular antigens within the sample by using a colloidal gold label, which can be visualized by TEM as small, electron‐dense objects. The sample used for antigen detection could be prepared from live virus (see Support Protocol 1), inactivated virus, viral‐like particles, purified proteins, or viral‐based vaccines. Samples should be well dispersed and dilute enough that individual particles on the grid are spaced sufficiently to unambiguously assign the target of the colloidal gold marker when visualized by TEM. This can be determined through negative staining of the sample (see Support Protocol 2). This protocol assumes that samples are stable without fixation. If necessary, a fixative agent such as paraformaldehyde can be used after applying samples to the grid before proceeding to incubation with antibodies. Antigens are labeled first with primary antibody targeting the antigen of interest and then with colloidal gold–conjugated secondary antibodies. Colloidal gold–conjugated protein A or protein G can be used as an alternative to colloidal gold–conjugated secondary antibody. Another alternative would be to use a colloidal gold–conjugated primary antibody, in which case no secondary antibody is needed. All procedures are carried out at room temperature.

### Materials

Viral particulate sample (see Support Protocol 1 for preparing live virus)
Distilled water
Blocking buffer (see recipe)
Primary antibody targeted to antigen of interest
Primary antibody targeted to irrelevant antigen (in same species)
Wash buffer (see recipe)
Colloidal gold–conjugated secondary antibody targeted to species of primary antibodies (e.g., Aurion, SPI Supplies, or Nanoprobes)
Negative staining solution (e.g., phosphotungstic acid [PTA]; see recipe)

**Figure 1:**
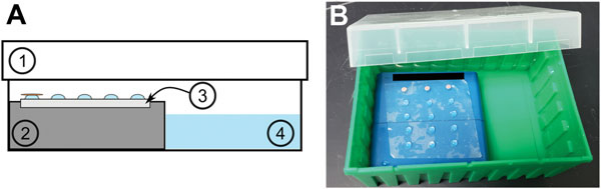
Example of humidified chamber setup. (**A**) Schematic of humidified chamber used for immunogold staining protocol. Chamber should have a lid (1) to prevent evaporation. There should be a raised platform (2), on which a piece of Parafilm can be placed (3). Incubation of grids with droplets of antibodies and/or buffers should be performed atop the Parafilm. Next to the platform should be a reservoir of water (4) that provides humidity to the chamber. Be sure to keep the grids on the Parafilm away from the water reservoir. (**B**) Humidified chamber used for the experiments described in this article. Chamber is made of an empty pipet tip box, 1.5‐ml microcentrifuge tube storage box, Parafilm, and water.

Parafilm
Carbon‐coated TEM grids, 300 or 400 mesh (e.g., Ted Pella, Quantifoil, Electron Microscopy Sciences)
Glass microscope slide
Glow discharge unit (e.g., PELCO easiGlow™ Glow Discharge Cleaning System, Ted Pella)
Self‐locking, fine‐point forceps
Whatman no. 1 filter paper
Humidified chamber (see Fig. 1A,B)
Grid storage box (e.g., Ted Pella, Electron Microscopy Sciences)
Transmission electron microscope

### Viral particulate deposition

1 Cut a piece of Parafilm from a roll, and place wax‐side down on a clean benchtop. Remove paper overlay.
  To ensure that the Parafilm adheres to the benchtop, a small amount of water can be placed onto the benchtop surface before placing the Parafilm on top.
2 *Optional*: Make carbon coating on grid(s) more hydrophilic using a glow discharge unit. To do so, place grid(s) carbon‐coated side facing upwards on a glass microscope slide, and insert slide into glow discharge unit. Treat grids for 25 sec at 15 mA.
3 Deposit viral suspension onto TEM grids to the carbon‐coated side, either by (a) floating the grid carbon‐side down on a drop of sample or (b) direct deposition of the viral sample onto the carbon‐coated side of the grid as follows:
  - For each grid, pipet 15 to 25 μl sample onto the Parafilm. Using forceps, gently place grid carbon‐coated side down onto the droplet of sample, being careful to not submerge the grid in the droplet. Allow sample to incubate with the grid for 1 min.
  - For each grid, use self‐locking forceps to hold the grid carbon‐coated side facing upward. Directly pipet 2 to 5 μl sample onto the surface of the grid, being careful to not bend the grid. Allow sample to incubate with the grid for 1 min.
4 Pipet 15 to 25 μl distilled water per grid onto the Parafilm.
5 Carefully pick up each grid by an edge using self‐locking forceps, and then remove excess liquid from each TEM grid using one of two methods:
  - Carefully wick away excess liquid from the grid surface by allowing the grid to touch the edge of filter paper. Ensure the grid does not dry completely.
  - Gently tap the side of the forceps against the edge of a gloved finger, allowing excess liquid to drop onto a piece of filter paper. Be careful not to use aggressive force, as doing so could cause the grid to fall off the forceps.
6 Before grids fully dry, transfer to water droplets prepared on the Parafilm, with carbon‐coated side down interacting with the water. Be careful to float each grid on the droplet, rather than submerging the grid into the droplet. Allow grids to remain on water droplets for ∼1 min.

### Antigen detection

7 In the humidified chamber, set up another piece of Parafilm, wax‐side down. Pipet 15 to 25 μl blocking buffer per grid. Leave enough room on the Parafilm for other droplets to be added later.
  The humidified chamber is a container with sealable lid with reservoir of water or water‐soaked paper towels (Fig. 1).
  The presence of Tween 20 in the blocking buffer creates a droplet that spreads on the Parafilm surface more than phosphate‐buffered saline (PBS) alone.
8 Using self‐locking forceps, pick up each grid by an edge, and gently remove excess liquid, as described in step 5. Immediately float each grid on a droplet of blocking buffer, carbon‐coated side down, before it has an opportunity to dry. Seal the lid of the humidified chamber, and allow grids to incubate with blocking buffer for 15 to 30 min.
9 For each grid, pipet 15 to 25 μl primary antibody of an appropriate dilution in blocking buffer onto the Parafilm in the humidified chamber. Carefully pick up each grid from the blocking buffer using self‐locking forceps, and gently remove excess liquid without allowing the grid to dry. Float grids in primary antibody, and seal the lid of the humidified chamber. Allow grids to incubate on the droplets of primary antibody for 1 to 2 hr.
  The antibody dilution will need to be empirically determined to identify the best antibody concentration, which has sufficient labeling with minimal background signal. Serial 10‐fold dilutions of antibody may be useful in determining the appropriate concentration. The authors have found that ideal antibody concentrations are usually in the range of 0.1 to 1 μg/ml. To evaluate the specificity of binding, control grids using an irrelevant antibody derived from the same species as the primary antibody of interest should be prepared at the same concentration as the determined concentration of the antibody of interest.
10 Wash each grid five times. To do so, for each grid pipet five droplets consisting of 15 to 25 μl wash buffer onto the Parafilm on the benchtop. Pick up grid from the blocking buffer with self‐locking forceps, ensuring that the grid does not get submerged into the droplet. Remove excess liquid, and then immediately transfer grid to the wash buffer, ensuring the grid floats on top of the droplet. Let the grid incubate for 3 min, and then remove excess liquid before transferring the grid to the next droplet of wash buffer. Repeat this process until each grid has been washed five times.
  If the room has very low humidity, the volume of wash buffer can be increased to prevent evaporation, or this step can be done in the humidified chamber.
11 While the grids are incubating with wash buffer, for each grid, pipet 15 to 25 μl colloidal gold–conjugated secondary antibody diluted in blocking buffer onto the Parafilm in the humidified chamber. After the wash step is complete, transfer each grid from the last droplet of wash buffer to a droplet of secondary antibody, removing excess liquid as described in step 5. Seal the humidified chamber, and allow grids to incubate for 60 min.
  There are numerous commercially available colloidal gold–conjugated antibodies (e.g., Aurion, SPI Supplies, Nanoprobes) that come in a variety of size ranges. The authors recommend using colloidal gold in the range of 5 to 25 nm.
  The colloidal gold–conjugated secondary antibody should be diluted in blocking buffer until the reddish color of the colloidal gold is barely detected by eye in the diluted solution. The authors recommend using a 1 in 20 dilution as a starting point. Before pipetting each droplet, the solution should be gently mixed, as the colloidal gold may settle in the tube.
12 Wash each grid five times in wash buffer using the method described in step 10. Then wash each grid three times in distilled water using the same method.
  It is important to wash the grid in water before proceeding to staining the grid, as salt ions in the PBS‐containing wash buffer may interfere with uniform deposition of the stain and result in electron‐dense crystals that obscure visualization of the sample.

### Negative staining

**Figure 2:**
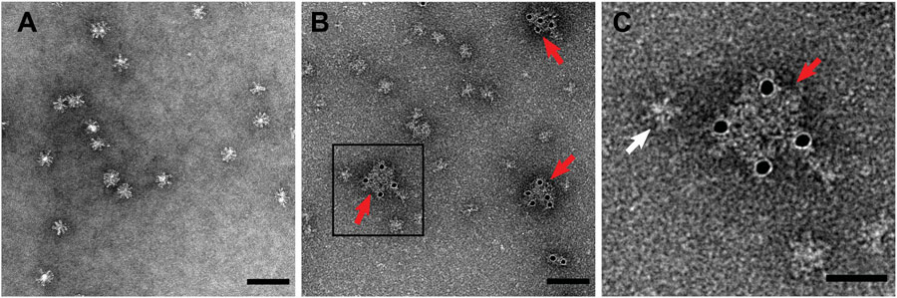
Immunoelectron microscopy of Flublok (Sanofi Pasteur), a commercially available influenza vaccine from 2017 to 2018 containing hemagglutinin from H1N1, H3N2, and influenza B viruses. (**A**) Negative stain of vaccine stained with 3% PTA. Scale bar, 100 nm. (**B**) Vaccine labeled for H1N1 hemagglutinin using polyclonal rabbit antibody and 10‐nm gold‐conjugated goat anti‐rabbit antibody (Aurion). Note that the complexes are small enough that antibody binding appears to alter the size of the complex (red arrows), as the antibodies can also be visualized. Scale bar, 100 nm. (**C**) Enlarged region from panel B showing complex containing H1N1 hemagglutinin antigen with immunogold labels (red arrow) compared with an unlabeled complex lacking H1N1 hemagglutinin antigens (white arrow). Scale bar, 50 nm.

13 Pipet 15 to 25 μl negative staining solution per grid to the Parafilm on the benchtop. Using self‐locking forceps, gently pick up the grid(s) from the distilled water, and remove excess liquid from the grid(s). Then transfer each grid to the surface of the negative staining solution for 30 to 60 sec.
14 *Optional*: Add an additional quick washing step after incubating the grid(s) with the negative staining solution if staining is too dense as determined by negative staining (see Support Protocol 2). To do so, while the grid(s) incubate with the negative staining solution, pipet 15 to 25 μl water for each grid onto the Parafilm. After the grid(s) have incubated with the staining solution, remove excess liquid as described in step 5. Then use self‐locking forceps to touch each grid to a droplet of water, and immediately remove excess distilled water.
15 Remove excess liquid from the grid(s), and touch the edge of each grid to a wedge of filter paper until the grid(s) appear dry.
  Some of the negative staining solution may be trapped between the arms of the self‐locking forceps during the blotting process. To remove this liquid, gently slide the pointed tip of the filter paper wedge between the arms of the forceps, being careful not to drop the grid in the process.
16 Allow grid(s) to air dry fully before inserting into a transmission electron microscope.
  Allowing the grids to fully dry will prevent damage to the sample and microscope contamination. Grids can be stored in a grid storage box in a chamber with desiccant to accelerate the speed of drying.
17 Examine grid(s) by TEM operated at 80 kV or higher, using an objective aperture to increase contrast.
  The colloidal gold will appear as small, electron‐dense (black) dots located near the antigen of interest in the microscope. See Figure 2 for an example of a viral vaccine sample (Fig. 2A) with immunogold labeling (Fig. 2B,C).

## STAINING FOR MULTIPLE TARGETS IN IMMUNOELECTRON MICROSCOPY USING GOLD‐CONJUGATED SECONDARY ANTIBODIES

Probing for multiple antigens with immunoelectron microscopy in a suspension of viral particulates can be a powerful tool for understanding molecular organization of a sample. However, care must be taken to ensure specific and distinct labeling of antigenic components. The same considerations are required as described in the Basic Protocol, including sample preparation and optional fixation. Of critical importance, the primary antibodies to be used for multiple labeling must be raised in different animal species (e.g., primary antibody to antigen 1 raised in mice, primary antibody to antigen 2 raised in rabbits, etc.). Colloidal gold–conjugated secondary antibodies must be specific to the species of primary antibodies, without species cross‐reactivity that may cause nonspecific labeling (e.g., antibody to mouse IgG raised in goat and antibody to rabbit IgG raised in goat but not antibody raised to rabbit IgG raised in mouse). Each secondary antibody should be conjugated to a different size of colloidal gold to distinguish the labels when imaged by TEM. To ensure that variability in colloidal gold particle size does not create ambiguity when identifying antigens, it is recommended to choose antibody conjugates with differences in colloidal gold size of >5 nm. The following protocol describes steps necessary to label multiple antigens using immunoelectron microscopy of a suspension of viral particulates, whether they are live or inactivated virus (see Support Protocol 1), viral‐like particles, purified proteins, or viral‐based vaccines. All procedures are carried out at room temperature.

### Additional Materials (also see Basic Protocol)

Primary antibodies targeting antigen of interest raised in different host species
Primary antibodies targeting irrelevant antigen raised in different host species
Colloidal gold–conjugated secondary antibody targeted to species of primary antibodies

**Figure 3:**
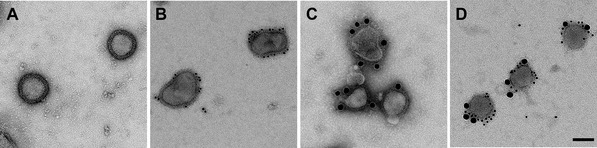
Immunoelectron microscopy of pandemic H1N1 influenza virus (A/California/04/09) produced in MDCK cells. (**A**) Negative stain of influenza virus stained with 3% PTA. (**B**) Virus labeled for hemagglutinin with C179 HA‐stem binding antibody and 10‐nm colloidal gold–conjugated goat anti‐mouse antibody. (**C**) Virus labeled for neuraminidase with polyclonal rabbit antibody and 25‐nm colloidal gold–conjugated goat anti‐rabbit secondary antibody. (**D**) Virus double labeled for hemagglutinin (10‐nm gold labels) and neuraminidase (25‐nm gold labels). Scale bar, 100 nm.

1 Apply viral suspension sample to TEM grid(s) as described in Basic Protocol step 3, either by direct application to the surface of the grid or by floating the grid on a droplet of sample.
2 Rinse grid by floating grid on a droplet of distilled water, followed by incubation with blocking buffer for 15 to 30 min in the humidified chamber, as described in the Basic Protocol.
3 Prepare primary antibody cocktail solution(s). Freshly mix multiple antibodies to the antigens of interest (or irrelevant antigens as control) raised in different host species, diluted in blocking buffer. Then pipet 15 to 25 µl antibody cocktail solution onto Parafilm in the humidified chamber. Carefully pick up grid(s) floating on blocking buffer, and gently remove excess liquid. Transfer grid(s) to float on the primary antibody cocktail solutions, carbon‐coated side down and interacting with the mixture. Allow grid to incubate for 1 to 2 hr.
  The antibody dilution will need to be empirically determined to identify the best antibody concentration, which has sufficient labeling with minimal background signal. Serial 10‐fold dilutions of antibody may be useful in determining the appropriate concentration, and the authors have found that ideal antibody concentrations are usually in the range of 0.1 to 1 μg/ml.
4 Wash each grid five times with wash buffer on the benchtop, as described in Basic Protocol step 10.
5 While grids are incubating with wash buffer, prepare a fresh cocktail solution of gold‐conjugated secondary antibodies diluted in blocking buffer. Pipet 15 to 25 μl secondary antibody cocktail solution for each grid onto the Parafilm in the humidified chamber. Transfer each grid from the last droplet of wash buffer to a droplet of the secondary antibody cocktail solution, making sure to remove excess liquid. Seal the humidified chamber, and allow grids to incubate for 60 min.
  Secondary antibodies can be raised in the same host species but should not be raised in the same species as any of the primary antibodies to avoid cross‐reactivity. Each secondary antibody should be conjugated to a different size of colloidal gold.
  As protein A and protein G can variably bind antibodies to numerous species, gold‐conjugated protein A and G are not recommended for multiple labeling of antigens. The cocktail solutions of secondary antibodies should be slightly reddish in color and well mixed before pipetting onto the Parafilm.
6 Wash each grid five times with wash buffer on the benchtop, and then wash three times in distilled water.
7 Pipet 15 to 25 μl negative staining solution per grid to the Parafilm on the benchtop. As described in the Basic Protocol, float each grid on negative staining solution for 30 to 60 sec.
8 *Optional*: Pipet 15 to 25 μl water for each grid onto the Parafilm. After grid(s) have incubated with the staining solution, remove excess liquid as described in Basic Protocol step 5. Then touch each grid to a droplet of water, and immediately remove excess distilled water.
  This wash step can be included if staining without a wash step is empirically determined to be too dense for sample alone (see Support Protocol 2).
9 Remove excess liquid from the grid(s), and touch the edge of each grid to a wedge of filter paper until the grid(s) appear dry. Allow grid(s) to air dry fully. Then examine grid(s) by TEM.
  See Figure 3 for an example of influenza virus immunogold labeled against two antigens (Fig. 3D), with 10‐nm and 25‐nm colloidal gold labels, compared with virus by only negative stain (Fig. 3A) and immunogold labeled against a single antigen (Fig. 3B,C).

## Support Protocol 1 VIRUS GROWTH AND PURIFICATION FOR USE IN ELECTRON MICROSCOPY

There are many aspects to the structural study of viruses including morphology, glycoprotein presence and distribution, and lipid bilayer organization. In order to visualize these viral characteristics, purified virions must be used. This protocol for viral propagation and purification using a mammalian cell culture system is reproducible, scalable, and lends itself to optimization based on the viral class being grown. The following protocol has been optimized for the propagation of influenza A viruses; however, the steps can be adapted to grow other viruses that require fusion for cellular entry (Hilleman, 2002). Influenza virus infection in Murine‐Darby canine kidney (MDCK) cells with optimized viral yield can lead to changes in such a generalized protocol (Ilyushina et al., 2012). Common changes could include the need for TPCK‐trypsin, incubation temperature, and the addition or absence of serum in the medium after virus adherence (Eisfeld, Neumann, & Kawaoka, 2014). Cell line clonality can also affect yield. In this protocol standard MDCK cells are used, but alternate types of MDCK cells are available, such as MDCK‐SIAT cells that can produce high‐yield viral infections (Lugovtsev, Melnyk, & Weir, 2013). Viral propagation in a permissive cell type is a crucial factor, and while influenza virus infects not only MDCK cells but also Vero cells, identifying the most appropriate cell line is virus specific. Implementing optimization conditions will have to be empirically determined based on the particular virus being studied. It is important to note that infectious virus can pose a safety concern and may require special handling according to its biosafety levels (BSLs). Consult with your institute's appropriate office concerning the safety and use of viral materials prior to beginning viral growth experiments. This should be addressed when preparing for viral growth and purification, as well as for subsequent immunoelectron microscopy experiments.

### Materials

Cell line for viral growth (e.g., MDCK cells; ATCC #FR‐926)
Dulbecco's Modified Eagle Medium (DMEM; e.g., Quality Biological)
DMEM containing 5% or 10% fetal bovine serum (FBS) and antibiotics (see recipes)
1× PBS, pH 7.4
0.05% trypsin‐EDTA
Influenza A virus source
2.5% TPCK‐trypsin (see recipe)

25‐cm^2^ and 75‐cm^2^ flasks (e.g., Corning)
37°C humidified incubator with 5% CO_2_
Inverted light microscope
15‐ml conical tube
Centrifuge

### Host cell preparation

1 Thaw a new vial of MDCK cells, and add to a 25‐cm^2^ flask with 5 ml prewarmed DMEM containing 10% FBS and antibiotics using sterile technique in a biological safety cabinet. Incubate flask in a 37°C humidified incubator with 5% CO_2_.
2 When cells have reached ∼90% confluency, aspirate medium and wash monolayer with 3 ml PBS. Aspirate PBS and add 1 ml of 0.05% trypsin‐EDTA. Rock flask back and forth to completely trypsinize the monolayer.
  The cells will start to slough off the bottom of the flask upon the addition of trypsin.
  The time it takes to completely trypsinize a monolayer can vary with density of the monolayer as well as age of the cells. The flask with trypsin‐EDTA can be left at room temperature in the biological safety cabinet or returned to the incubator at 37°C to aide in trypsinization. Monitor the flask, and as soon as the cells start to slough off the bottom of the flask, proceed to step 3. The detached cells will give a cloudy appearance to the trypsin‐EDTA, and when completely detached, the cells can be observed under an inverted light microscope as round and floating.
3 Once all cells have detached from the bottom of the flask, add 12 ml DMEM containing 5% FBS and antibiotics with a serological pipet to quench the trypsinization, and pipet up and down multiple times to disrupt any cell clumps that may be present.
4 Add cells to a 75‐cm^2^ flask, and incubate in a 37°C humidified incubator with 5% CO_2_.
  Millions of cells will likely be transferred to the 75‐cm^2^ flask during this step, though an exact cell count from the 25‐cm^2^ flask is not necessary since the entire volume will be used to seed the 75‐cm^2^ flask and since the cell growth surface area is being expanded 3‐fold.
5 Monitor cell growth by observing the monolayer using an inverted light microscope. When monolayer reaches 85% to 90% confluency, proceed to the next step.
6 Aspirate growth medium from the 75‐cm^2^ flask. Gently rinse monolayer with 5 ml prewarmed DMEM without FBS or antibiotics. Aspirate rinse, being careful to not let the monolayer dry, and add 3 ml DMEM without FBS or antibiotics.

### Viral infection

7 Add multiplicity of infection (MOI) of 0.01 virus source to 200 μl DMEM without FBS or antibiotics. Add complete volume to the flask, and rock to cover the flask, making sure there is full coverage so that the monolayer does not dry.
  Infecting monolayers at an MOI >0.01 plaque forming units/ml (PFU/ml) is a common parameter for influenza virus to prevent multiple viruses from infecting a single cell. For other viruses, a different MOI may be needed. Determining MOI via plaque assays or TCID_50_ are beyond the scope of this protocol. As this protocol has been optimized using in‐house viral stocks, 2 μl virus stock in 200 μl DMEM has been sufficient for infecting a naïve monolayer.
8 Incubate flask in a 37°C humidified incubator with 5% CO_2_ for 1 hr to absorb the virus to the cell surface. During this hour, periodically and briefly remove flask from the incubator, and rock flask back and forth to distribute the volume of liquid over the monolayer.
9 After 1 hr, remove flask and add 3 ml DMEM containing 5% FBS, antibiotics, and 0.125 μl of 2.5% TPCK‐trypsin. Do not aspirate the previous inoculum.
  There should be a final volume of ∼6 ml.
  TPCK‐trypsin denotes a treatment of trypsin to inhibit contaminating chymotryptic activity. TPCK‐trypsin is used in influenza virus growth to cleave and activate influenza‐specific glycoproteins but can be omitted for other virus preparations. As is the case with influenza virus, it may be necessary to use reagents specific for a particular virus being produced, and these reagents should be investigated prior to beginning the protocol.
10 Place flask back in the incubator using the same conditions as described in step 1.
11 Monitor infection for cytopathic effect (CPE) using an inverted light microscope.
  CPE is observed when the cells appear rounded, clumped, and/or start to release from the bottom of the flask.
12 When ∼80% to 90% of the monolayer is showing CPE, harvest medium and place into a 15‐ml conical tube.
13 Centrifuge medium 20 min at 3200 to 3700 × *g*, 4°C, to pellet cellular debris.
14 Remove supernatant from the pellet.
  This supernatant contains the virus that will be pelleted.
15 Centrifuge supernatant 3.5 hr at 42,000 × *g*, 4°C. Remove and discard supernatant from the white pellet visible on the tube wall. Resuspend pellet in 75 to 100 μl PBS.
16 Visualize virus using negative staining TEM as described in Support Protocol 2.
  Figure 3A demonstrates a pandemic H1N1 influenza A virus sample (A/California/04/09) purified via this method as imaged by negative staining TEM.

## Support Protocol 2 NEGATIVE STAINING ELECTRON MICROSCOPY OF VIRAL SUSPENSION SAMPLES

Negative staining TEM is a common method for screening samples to confirm successful sample preparation and to identify structural morphology. This is an important first step in identifying and analyzing the morphology of a particulate sample prior to beginning an immunogold staining experiment. The steps of this protocol closely mimic the final preparation of an immunogold‐labeled grid after antibody incubation, as immunogold‐labeled samples must also be stained using a negative staining solution to detect the morphology of the sample.

In this technique, a particulate sample is dispersed onto a carbon‐coated grid, and then a staining solution that surrounds the particles is applied. In the microscope, the negative stain appears dark due to the density of the heavy metal stain, while the particles appear light in color where the stain has been excluded. An ideal particulate sample would be highly pure and concentrated enough that multiple viral particulates can be imaged in a single view at the preferred magnification, while dilute enough that individual particles are separated rather than clustered together. Samples should ideally be suspended in a low‐salt buffer, as salts can interact with some negative staining solutions resulting in the appearance of opaque crystals on the grid.

Multiple negative staining solutions can be used to visualize viral particulate samples, including PTA, uranyl acetate (UA), and methylamine tungstate, among others. Each stain has advantages and disadvantages based on its grain size, which affects resolution, electron density, and contrast. Multiple stains may need to be tested, but in general PTA often produces good results for a viral particulate sample. PTA is very commonly used in studying viruses as it can be used at neutral pH and is compatible with many viral particulate samples. However, PTA does not produce as much contrast or as high of resolution as some other stains and may interact with some lipoproteins (Rames, Yu, & Ren, 2014). UA has very good contrast and resolution and acts as a fixative for samples. However, UA solutions are acidic (pH 4 to 5) and precipitate at neutral pH; therefore UA should not be used as a staining solution for samples that are unstable in acidic conditions. Methylamine tungstate has good resolution but is less electron dense than UA and therefore does not have as much contrast. However, it is near‐neutral pH (pH 6.8) and does not disrupt lipoprotein architecture like PTA.

### Materials

Distilled water
Negative staining solution: PTA (see recipe), UA (see recipe), or methylamine tungstate (e.g., Nano‐W®, NanoProbes)
Viral particulate sample (see Support Protocol 1 for preparing live virus)

Carbon‐coated TEM grids, 300 or 400 mesh (e.g., Ted Pella, Quantifoil, Electron Microscopy Sciences)
Glass microscope slide
Glow discharge unit (e.g., PELCO easiGlow™ Glow Discharge Cleaning System, Ted Pella)
Parafilm
Self‐locking forceps
Whatman no. 1 filter paper
Grid storage box (e.g., Ted Pella, Electron Microscopy Sciences)
Transmission electron microscope

1 *Optional*: Make the carbon coating on the grid(s) more hydrophilic using a glow discharge unit. To do so, place grid(s) carbon‐coated side facing upwards on a glass microscope slide, and insert slide into the glow discharge unit. Treat grids for 25 sec at 15 mA.
2 Prepare a piece of Parafilm on a clean benchtop as described in Basic Protocol step 1. For each grid of sample being negative stained, pipet 15 to 25 μl distilled water and 15 to 25 μl negative staining solution onto the Parafilm.
3 Apply viral particulate sample to carbon‐coated side of the TEM grid(s) either by direct application to the grid or by floating the grid on a droplet of sample, as described in Basic Protocol step 3.
4 Carefully pick up each grid by an edge using self‐locking forceps, and remove excess liquid from each TEM grid as described in Basic Protocol step 5.
5 Without allowing grid(s) to fully dry, transfer to the water droplets prepared on the Parafilm with the carbon‐coated side facing down interacting with the water. Be careful to float each grid on the droplet without submerging it. Allow grid(s) to remain on the water droplets for ∼1 min.
6 Gently pick up each grid from water droplet using forceps, and remove excess liquid from the grid. Then transfer each grid to the surface of a droplet of negative staining solution for 30 to 60 sec.
7 *Optional*: Add an additional quick washing step after incubating each grid with the negative staining solution if staining is too dense. To do so, pipet 15 to 25 μl water onto the Parafilm per grid. After removing grid(s) from the negative staining solution, use self‐locking forceps to touch each grid to the droplet of water, and immediately remove excess liquid as described in Basic Protocol step 5.
8 Remove excess liquid from each grid, and then touch its edge to a wedge of filter paper until grid(s) appear dry. Remove excess liquid that may be trapped between the arms of forceps with filter paper, as well.
9 Allow grid(s) to air dry.
  The grid can be stored in a grid storage box in a chamber with desiccant to accelerate the speed of drying.
10 Examine grids by TEM.
  For examples of successful negative staining TEM using PTA, see Figures 2 and 3.

## REAGENTS AND SOLUTIONS

### Blocking buffer

99 ml PBS, pH 7.4 (commercially available)
100 μl Tween 20
1 ml 30% bovine serum albumin (BSA), IgG free (e.g., Sigma‐Aldrich)
Mix well
Store at 4°C for up to 6 months

### DMEM containing 5% FBS and antibiotics

500 ml DMEM (e.g., Quality Biological)
25 ml heat‐inactivated, low IgG FBS (e.g., Thermo Fisher Scientific)
5 ml penicillin‐streptomycin (10,000 U/ml penicillin, 10 mg/ml streptomycin; e.g., Sigma‐Aldrich)
Mix well
Store at 4°C for up to 4 months

To heat inactivate the FBS stock, heat serum in a 56°C water bath for 30 min.

### DMEM containing 10% FBS and antibiotics

500 ml DMEM (e.g., Quality Biological)
50 ml heat‐inactivated, low IgG FBS (e.g., Thermo Fisher Scientific)
5 ml penicillin‐streptomycin (10,000 U/ml penicillin, 10 mg/ml streptomycin; e.g., Sigma‐Aldrich)
Mix well
Store at 4°C for up to 4 months

To heat inactivate the FBS stock, heat serum in a 56°C water bath for 30 min.

### Negative staining solution: phosphotungstic acid (PTA)

Prepare a 1% to 3% (w/v) solution of PTA (e.g., Electron Microscopy Sciences, Ted Pella, SPI Supplies) in water, and adjust pH to 7.0 using NaOH. PTA is stable for months at room temperature.

PTA can be used at acidic pH but is most commonly used at pH 7.0.

### Negative staining solution: uranyl acetate (UA)

Prepare a 1% to 3% (w/v) solution of UA (e.g., Electron Microscopy Sciences, Ted Pella, SPI Supplies) in water. Then filter solution with a 0.22‐μm filter that has been prerinsed with distilled water. Stored in the dark at 4°C. If properly stored, UA is stable for months.

Consult with your institute's appropriate safety office concerning the use and proper disposal of materials related to uranyl stains.

### TPCK‐trypsin, 2.5%

100 ml 1 mM HCl (prepared in cell culture–grade water)
2.5 g TPCK‐trypsin (e.g., Sigma‐Aldrich)
Mix well
Filter using a 0.45‐µm filter
Aliquot and store at −20°C for up to 1 year

Filter using either a sterile syringe filter or a vacuum unit.

Generate small‐volume aliquots as trypsin is sensitive to freeze/thaw cycles; thus any unused volume of a thawed aliquot is best discarded.

### Wash buffer

100 ml PBS, pH 7.4 (commercially available)
100 μl Tween 20
100 μl 30% BSA, IgG free (e.g., Sigma‐Aldrich)
Mix well
Store at 4°C for up to 6 months

## COMMENTARY

### Background Information

TEM has been used in molecular virology for 80 years, when tobacco mosaic virus was first visualized in an electron microscope (Kausche, Pfankuch, & Ruska, 1939). The improved resolution of electron microscopes over traditional light microscopes transformed numerous aspects of virology. Samples of viral particles suspended in liquid samples have been imaged by negative staining to identify new viruses such as adenovirus, hepatitis B, norovirus, and many others, either from cell culture systems or clinical samples (Kapikian, 2000; Roingeard, 2008). Beyond identification, negative staining was used to classify viruses and diagnose patients. Ultrathin sections of tissues or cellular samples have been imaged for elucidating viral pathology pathways (Marsh & Helenius, 2006; Wilson, Pedroza, Beuerman, & Hill, 1997). Immunogold labeling has played a key role in localizing antigens and understanding certain viral‐host interactions (Cristea et al., 2006; Hopley & Doane, 1985).

The field of TEM has evolved since the early days, and new advances in cryoelectron microscopy, for which the 2017 Nobel Prize in Chemistry was awarded, have resulted in numerous high‐resolution virus structures (Cressey & Callaway, 2017; Jiang & Tang, 2017). Nevertheless, cryoelectron microscopy requires complex sample preparation, substantial investment of equipment, and intense computational analysis. The techniques that first made TEM such a powerful tool in virology can still provide valuable information. Liquid samples can be dried to the grid and imaged using negative staining or immunogold staining to reveal structural information and antigen presentation of a sample more quickly and with less investment than cryomethods, as outlined in this article. Ultrathin sections of clinical samples are still used in studies of viral cell entry pathways, assembly and disassembly, and maturation (Archer et al., 2017; Bamunusinghe, Chaturvedi, Seo, & Rao, 2013; Ou, Deerinck, Bushong, Ellisman, & O'Shea, 2015; Wang et al., 2017).

### Critical Parameters

There are specific safety concerns to be addressed when preparing immunogold‐labeled grids and disposing of them. Certain samples, including some live viruses, will require special handling according to their BSLs due to their infectious nature. For example, pandemic H1N1 influenza virus requires BSL‐2 handling. Additionally, some negative staining solutions require proper disposal. For example, UA is toxic and contains trace levels of radioactivity, and thus it must be disposed of in radioactive waste. These concerns should be addressed when designing experiments.

Many variables must be properly optimized to successfully prepare immunogold‐labeled grids for TEM (see Troubleshooting). It is also important to note that the order in which variables are optimized can greatly affect productivity. It is highly recommended to first optimize negative staining of unlabeled samples, since interpretation of labeling requires good contrast from negative stain. This technique will also reveal basic morphology of the sample for comparison with immunogold‐labeled samples. Second, the antibody concentrations should be optimized to ensure successful labeling of the antigen of interest while maintaining low background labeling. Serial antibody dilutions may be useful to identify an appropriate concentration.

Controls are essential to interpreting results from an immunoelectron microscopy experiment to account for nonspecific labeling and to account for morphology changes that may arise due to the presence of antibodies. Preparing the necessary controls requires additional grids to be made with different antibody combinations, which should be performed at the same time as the experimental grids. A control grid should be prepared with a primary antibody to an irrelevant antigen raised in the same host species as the experimental antibody, followed by the same secondary antibody as used for the experimental grid. This control will account for nonspecific labeling to ensure specific binding to the target antigen. If available, using an alternative on‐target antibody from a different species could be an excellent method to validate immunoelectron microscopy results. To control for changes in the appearance of a complex when labeled with antibody, another control grid should be prepared with the experimental primary antibody combined with a secondary antibody that is not conjugated with colloidal gold. This control will indicate how morphology may change in the presence of antibodies. For example, small particulates may appear larger in the presence of antibodies, as the antibodies themselves may appear by negative stain. This can be seen for the Flublok (Sanofi Pasteur) viral vaccine candidate in Figure 2, in which the immunogold‐labeled complexes appear larger than nonlabeled complexes. If performing immunoelectron microscopy targeting multiple antigens simultaneously (Alternate Protocol), cross‐reactivity between antibodies should be tested. For example, when an experiment is designed to label two antigens, one with a mouse antibody and one with a rabbit antibody, two additional control grids are needed. In the first, the mouse primary antibody should be used followed by a gold‐conjugated secondary antibody targeting rabbit. The opposite should be prepared for the second grid.

TEM imaging parameters are critical to successfully visualize immunogold‐labeled samples and interpret data. It is important to ensure the microscope is well aligned, grids are imaged slightly under focus, and an appropriate magnification is chosen such that multiple particulates can be visualized in one field of view while still detecting details of the particles. Specific operating instructions for TEM are dependent on the microscope used and are beyond the scope of this section.

### Troubleshooting

Immunoelectron microscopy has multiple steps at which problems can occur. Unfortunately, for any given grid, the errors often do not become obvious until the very end of the procedure, when grids are imaged by TEM. The most common problems that occur are poor sample quality, lack of labeling or nonspecific labeling, and poor visualization of sample.

Sample quality should be assessed through negative staining of the sample alone prior to beginning immunogold‐labeling experiments. Samples must be properly optimized for concentration and distribution on the grid. The concentration of viral particulate, whether virus, purified viral protein, or viral vaccine, should be low enough to resolve individual particles but high enough that multiple particles can be viewed in a single field of view at the preferred magnification of the microscope. Additionally, particles should be well dispersed to successfully identify with which particle the colloidal gold labels are associated. The ideal sample should be relatively free of contamination and thus may require purification steps. When preparing grids for imaging, if a sample does not adhere well to the grid, the grid can be pretreated with a wetting agent, such as poly‐l‐lysine, or can be made more hydrophilic using a glow discharge unit prior to application of the sample. If structural details of the sample cannot be resolved in TEM prior to immunogold labeling, the negative stain grain size may be too large, and a stain with higher resolution may be required. For example, UA has a smaller grain size and therefore higher resolution than PTA. Still, the uncertainty in the location of an epitope relative to the gold marker far exceeds the granular resolution of all commonly used negative staining solutions. Differences between common negative staining solutions are discussed in Support Protocol 2. Upon immunogold labeling, if multiple particles are proximal to a gold marker and it is unclear which particle has been labeled, sample concentration may be too high. In this case the sample should be diluted, preferably with a low‐salt buffer. The appearance of the sample may be altered by immunogold staining due to the multiple wash steps in the presence of mild detergent (e.g., if the sample has a membrane that may become deformed). In this case the sample can be fixed with a cross‐linking agent, such as paraformaldehyde or glutaraldehyde, prior to incubation with antibody.

**Figure 4:**
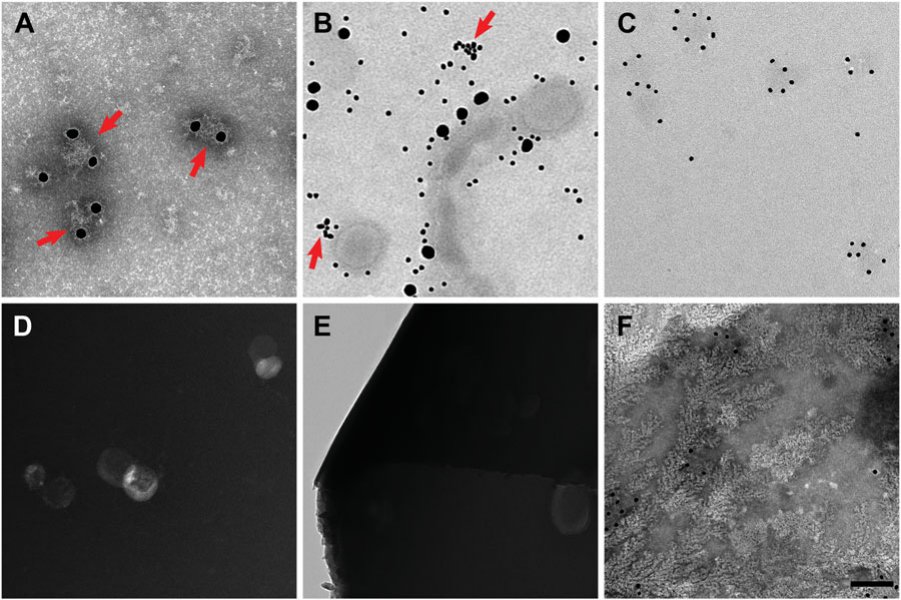
Troubleshooting errors when preparing immunogold‐labeled or negative‐stained TEM grids. (**A**) 25‐nm gold labels cannot fully label Flublok influenza vaccine antigens or fully surround the complexes compared with 10‐nm gold‐labels shown in Figure 1B, possibly due to steric hindrance from neighboring gold‐labeled antibodies. Nevertheless, the labeled complexes appear larger because primary antibodies were successfully able to bind (red arrows). (**B**) Nonspecific background labeling of pandemic H1N1 influenza A virus prevents successful interpretation of antigen presence. Red arrows indicate antibody aggregates, which may also limit data interpretation. (**C**) Even in conditions of poor‐contrast negative staining, the presence of Flublok antigens is evidenced by 10‐nm gold‐labeled H1N1 hemagglutinin. (**D**) Dense negative staining of pandemic H1N1 influenza A virus makes samples appear opaque; thus, interpretation of morphology is challenging. (**E**) Heavy staining can lead to broken carbon on the grid of pandemic influenza A virus. (**F**) Fractal‐like artifacts due to interactions between phosphate‐containing buffer and PTA negative staining solution make analysis of the Flublok sample challenging. Scale bar, 100 nm.

Poor labeling may be caused by multiple factors. One cause could be if there is a very low concentration of antigen. It could be also be caused by an antibody that may have lost its binding ability, or there may be incompatibility between antibody and blocking solution or subsequent negative staining solution. Antibody binding to the sample should be verified in this case by another means, such as through an enzyme‐linked immunosorbent assay (ELISA). Antibody concentration could also be increased. If gold labeling saturates at a level much lower than expected even with sufficiently high antibody concentrations (Fig. 4A), it could be that antigens are very close together, and there may be steric hindrance between neighboring antibodies and/or their gold labels that prevents additional gold‐conjugated antibodies from binding. In this case, it may be necessary to use a smaller size of colloidal gold–conjugated antibody (e.g., 10‐nm gold‐labeled antibody instead of 25‐nm gold‐labeled antibody). If labels are not only binding the antigen of interest but also nonspecifically present in the background (Fig. 4B), wash steps could be inadequate, in which case additional wash steps can be added. Alternatively, antibody concentration could be too high, and primary and/or secondary antibodies may need further dilution. If the gold labels appear to be clustered irrelevant of antigen presentation (Fig. 4B, arrows), this could be due to natural amplification of secondary antibodies bound to primary antibodies, in which case further dilution of secondary antibody can be performed, or to clumped primary antibody, in which case a fresh sample of primary antibody may be needed. An alternate solution to optimizing two sets of antibodies would be to use a colloidal gold–conjugated primary antibody, which then precludes the need to use any secondary antibody solutions. Doing so eliminates concerns of nonspecific binding by the secondary antibody, although nonspecific binding of the primary antibody may still be an issue. However, few colloidal gold–conjugated primary antibodies exist on the market, so it may be necessary to chemically conjugate colloidal gold to an antibody of interest, which is beyond the scope of this article.

Poor visualization of the sample may be caused by too light or too heavy negative staining, as a result of positive staining (Ackermann, 2013), or due to broken carbon on the grid itself. General negative staining procedures should be addressed prior to beginning immunogold labeling through negative staining of sample alone. Successfully negative‐stained particles should appear light in color, with the stain of the carbon support appearing as dark. If colors have inverted, where the particles appear as dark on a light background, positive staining has occurred, and negative staining conditions may need to be reevaluated. Possible solutions include altering the negative staining solution used or modifying the concentration of the stain to alter the amount of stain present on the grid. If staining is too weak (Fig. 4C), it will be difficult to identify and analyze particulates on the grid; in this case, the concentration of negative stain can be increased. If staining is too dense, this could lead to difficulties in visualizing the sample on the grid as the sample may appear opaque or have poor contrast compared to the background (Fig. 4D). To remedy this, a wash step can be performed after application of negative staining solution, or the concentration of negative stain can be lowered.

Heavy staining could also lead to broken carbon on the grid. Grids with broken carbon would appear empty under the microscope, as if no stain or sample is present (Fig. 4E, left). Another cause of broken carbon could be due to using old grids, as carbon becomes more fragile over time. The carbon could also have become brittle if treated in a glow discharge unit multiple times or for a very long incubation. In these cases, new grids may need to be acquired. Rough handling of the grid may lead to breaking of the carbon. In this case, using grids that have formvar plastic support along with carbon may be beneficial. If the grid appears to have patches of irregular particles under the microscope, it could be because the grid dried during preparation. It is critical to keep the grid hydrated throughout the entire immunogold staining procedure. It may be necessary to increase droplet size of liquids on Parafilm or perform all steps in a humidified chamber (Fig. 1). Patchy grids could also be caused by interactions between buffer and the negative staining solution (Fig. 4F). PTA interacts with phosphate buffers to produce fractal‐like artifacts, while UA interacts with phosphate buffers to create crystal‐like artifacts. In this case additional wash steps with distilled water may be necessary before applying the negative staining solution to the grids.

### Understanding Results

#### Statistical analyses

Immunogold labeling of viral particulate suspensions is often interpreted through qualitative and visual means. Statistical analyses, such as calculating and analyzing the number of gold labels associated with a specific particulate, can be done but are dependent on the sample being studied and therefore beyond the scope of this article. When doing so, however, it is important to keep in mind that antibodies may not fully bind to the antigen of interest or that not every primary antibody will be bound by a colloidal gold–conjugated secondary antibody, as these will depend on steric hindrance, antibody concentrations, and binding rates. Any quantitation that is done must also take into consideration the amount of background labeling that appears when colloidal gold labels are found on the support film with no viral particles present. The ratio of specifically to nonspecifically bound gold labels is important for estimating the error of an immunogold labeling experiment. To calculate the ratio, collect multiple TEM micrographs at the desired magnification, and determine an average number of nonspecific colloidal gold particles over all of the micrographs. Compare that to the number of antigen‐bound colloidal golds. An acceptable ratio for binding specificity during immunogold labeling would be at least 10:1, otherwise erroneous interpretations of the labeling are likely.

#### Typical results

Antigens of interest can be identified using colloidal gold labels after immunoelectron microscopy methods. The figures provide examples of successful labeling of hemagglutinin glycoproteins, both on the commercially available Flublok (Sanofi Pasteur) influenza vaccine sample (Fig. 2) and live influenza A H1N1 virus (Fig. 3). Immunogold labels outline the edges of both samples, as glycoproteins protrude on the exterior of both samples with minimal background labeling. This is in stark contrast to negative staining microscopy of the same samples, which displays the morphology of virus and vaccine samples without colloidal gold labels. Note that the background of the immunogold‐labeled sample appears to be fully coated with protein, compared with the pure carbon background of sample that is simply negative stained. This is because blocking with BSA leads to nonspecific albumin deposition on the grid, which can be visualized in the background but does not interfere with labeling.

### Time Considerations

Immunogold staining requires several hours to complete due to many long incubations and repeated careful grid handling. It is therefore recommended to do several grids in parallel to maximize the time cost, as preparing additional grids does not significantly lengthen the time needed. The authors recommend always performing negative staining TEM on any samples before immunogold staining protocols are begun, as negative staining can be done relatively quickly and can be done to ensure the sample conditions are optimized for immunogold staining.
